# Supplementary material for: Chaperone condensates buffer the heat shock response against pleiotropic inputs
Source: bioRxiv. 2026 Jul 30:2026.07.30.740154. Preprint. [Version 1] doi: 10.64898/2026.07.30.740154 (PMC13484205; doi:10.64898/2026.07.30.740154)
Supplement: Supplementary file 1 [file NIHPP2026.07.30.740154v1-supplement-1.pdf]

bioRxiv preprint doi: https://doi.org/10.64898/2026.07.30.740154; this version posted July 30, 2026. The copyright holder for this preprint (which was not certified by peer review) is the author/funder, who has granted bioRxiv a license to display the preprint in perpetuity. It is made available under a CC-BY 4.0 International license.

# Supplementary Figures

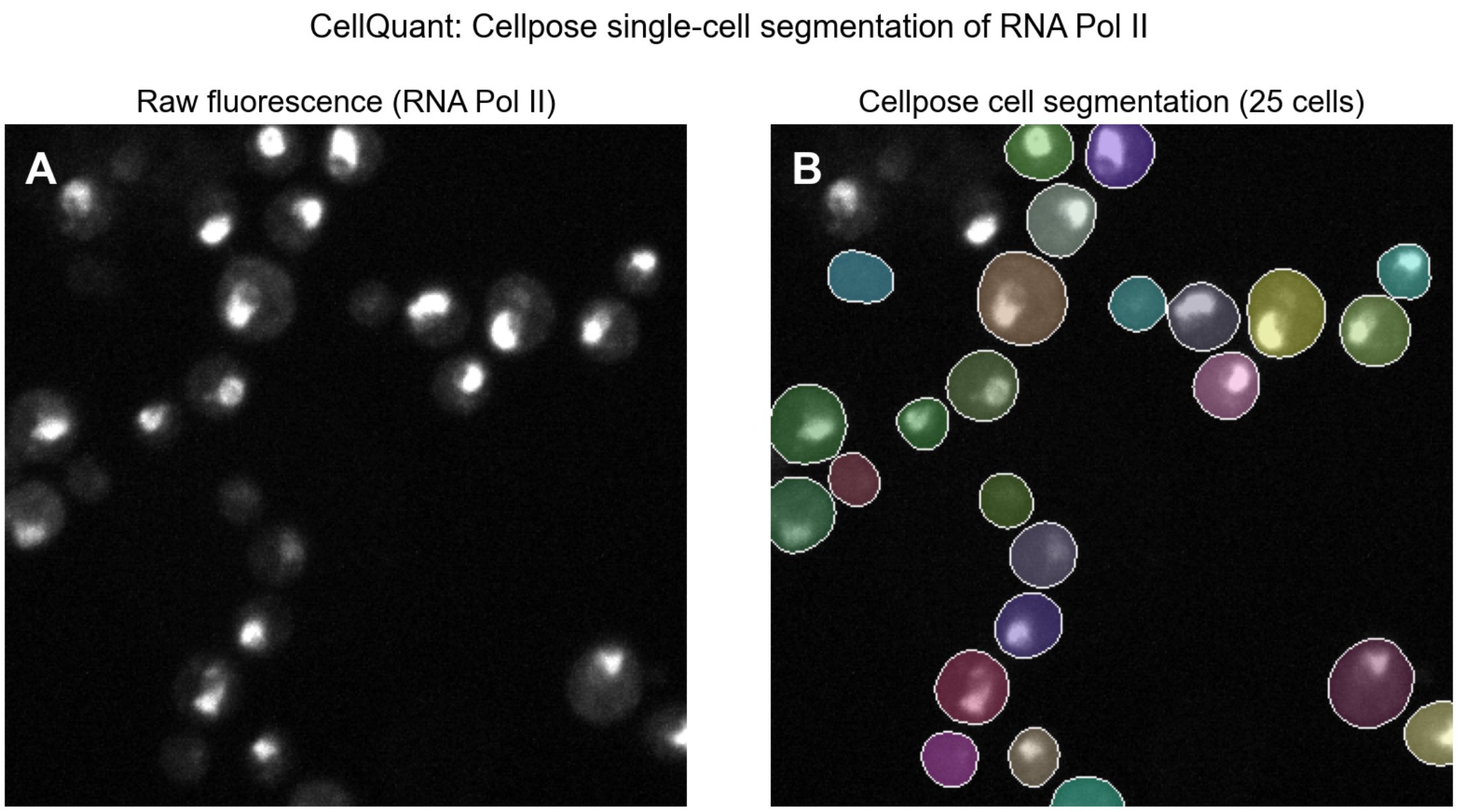


***Figure S1. Single-cell segmentation with the CellQuant pipeline.*** *Representative field of cells expressing endogenously tagged RNA polymerase II (Rpb3). (A) Raw fluorescence shown as a maximum intensity projection. (B) Whole-cell segmentation by Cellpose (Stringer et al. 2021): each detected cell is assigned a distinct label (random colors) and its boundary outlined in white. All condensation-index measurements in this study were computed cell-by-cell from CellQuant segmentations of images acquired identically across proteins and stress conditions (see methods). Scale bar, 5 µm.*

bioRxiv preprint doi: https://doi.org/10.64898/2026.07.30.740154; this version posted July 30, 2026. The copyright holder for this preprint (which was not certified by peer review) is the author/funder, who has granted bioRxiv a license to display the preprint in perpetuity. It is made available under a CC-BY 4.0 International license.

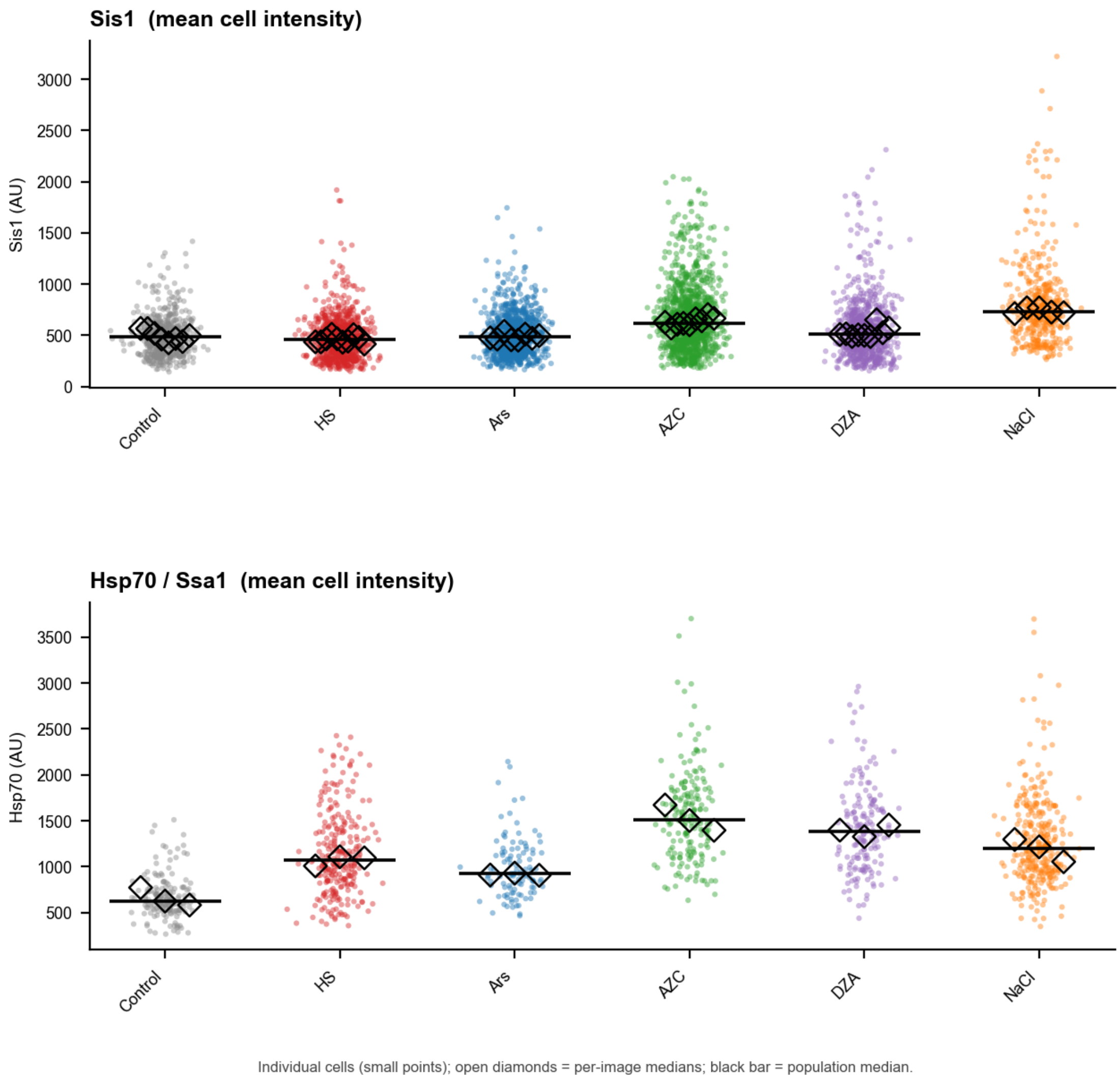


***Figure S2. Expression levels of Sis1 and Hsp70 across stresses.*** *Per-cell mean fluorescence intensity (a proxy for total cellular abundance) of endogenously tagged Sis1 and Hsp70 (Ssa1), quantified by CellQuant across the stress panel and displayed as superplots. Small points, individual cells; open diamonds, per-image medians; black bar, population median.*

bioRxiv preprint doi: https://doi.org/10.64898/2026.07.30.740154; this version posted July 30, 2026. The copyright holder for this preprint (which was not certified by peer review) is the author/funder, who has granted bioRxiv a license to display the preprint in perpetuity. It is made available under a CC-BY 4.0 International license.

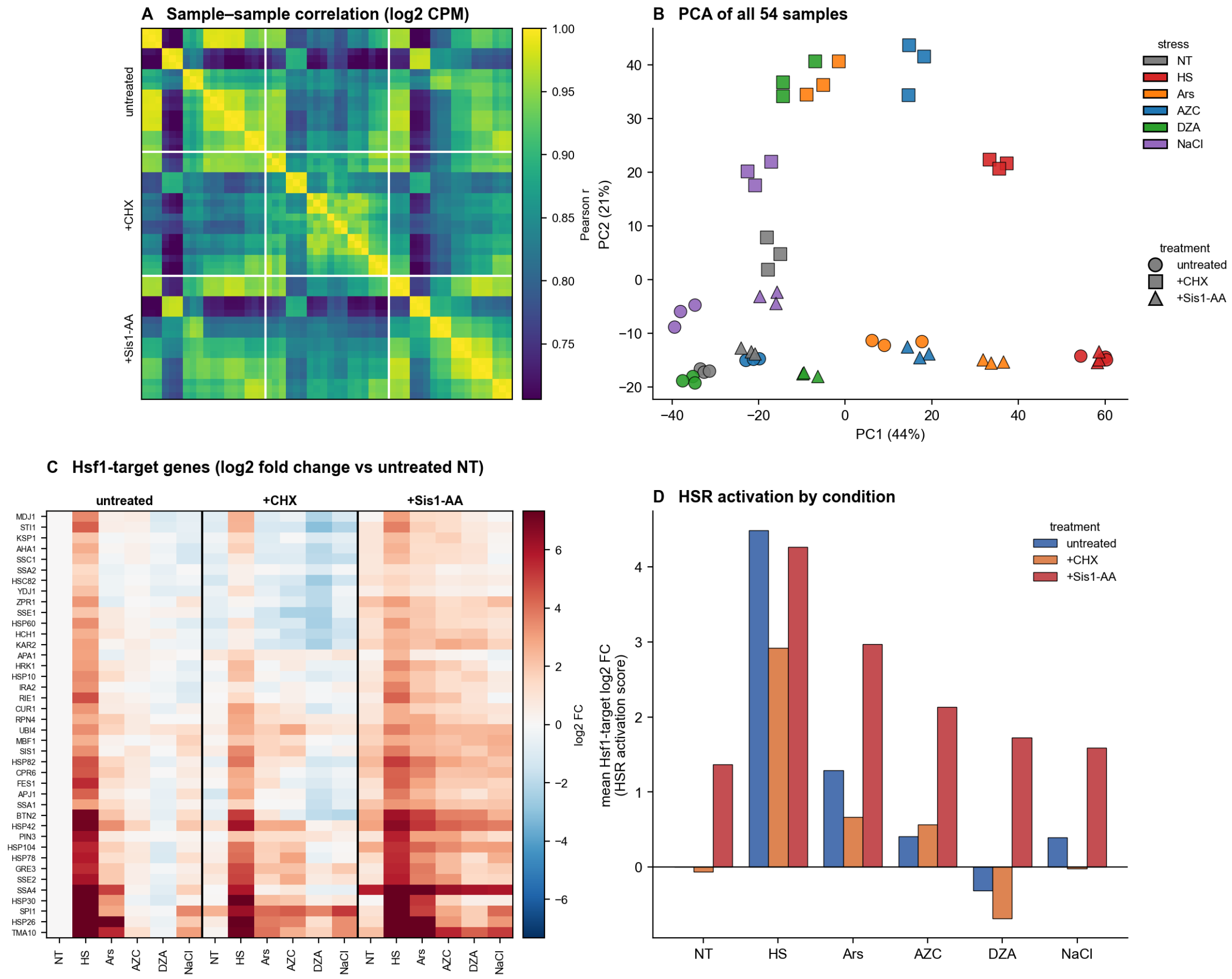


***Figure S3. Overview of the RNA-seq datasets.*** *(A) Pearson correlation between all 54 samples computed on* $log_2$ *CPM, ordered by treatment block (untreated, +CHX, +Sis1 anchor-away) and by stress; the block-diagonal structure confirms reproducibility among biological triplicates. (B) Principal-component analysis of all 54 samples (top 2000 most variable genes); color denotes stress and symbol denotes treatment. (C) Expression of Hsf1 target genes (rows) across the 18 conditions (mean of three replicates), shown as* $log_2$ *fold change relative to untreated NT and clustered by gene. (D) HSR activation score (mean* $log_2$ *fold change across the Hsf1 targets) for each stress, grouped by treatment.*

bioRxiv preprint doi: https://doi.org/10.64898/2026.07.30.740154; this version posted July 30, 2026. The copyright holder for this preprint (which was not certified by peer review) is the author/funder, who has granted bioRxiv a license to display the preprint in perpetuity. It is made available under a CC-BY 4.0 International license.

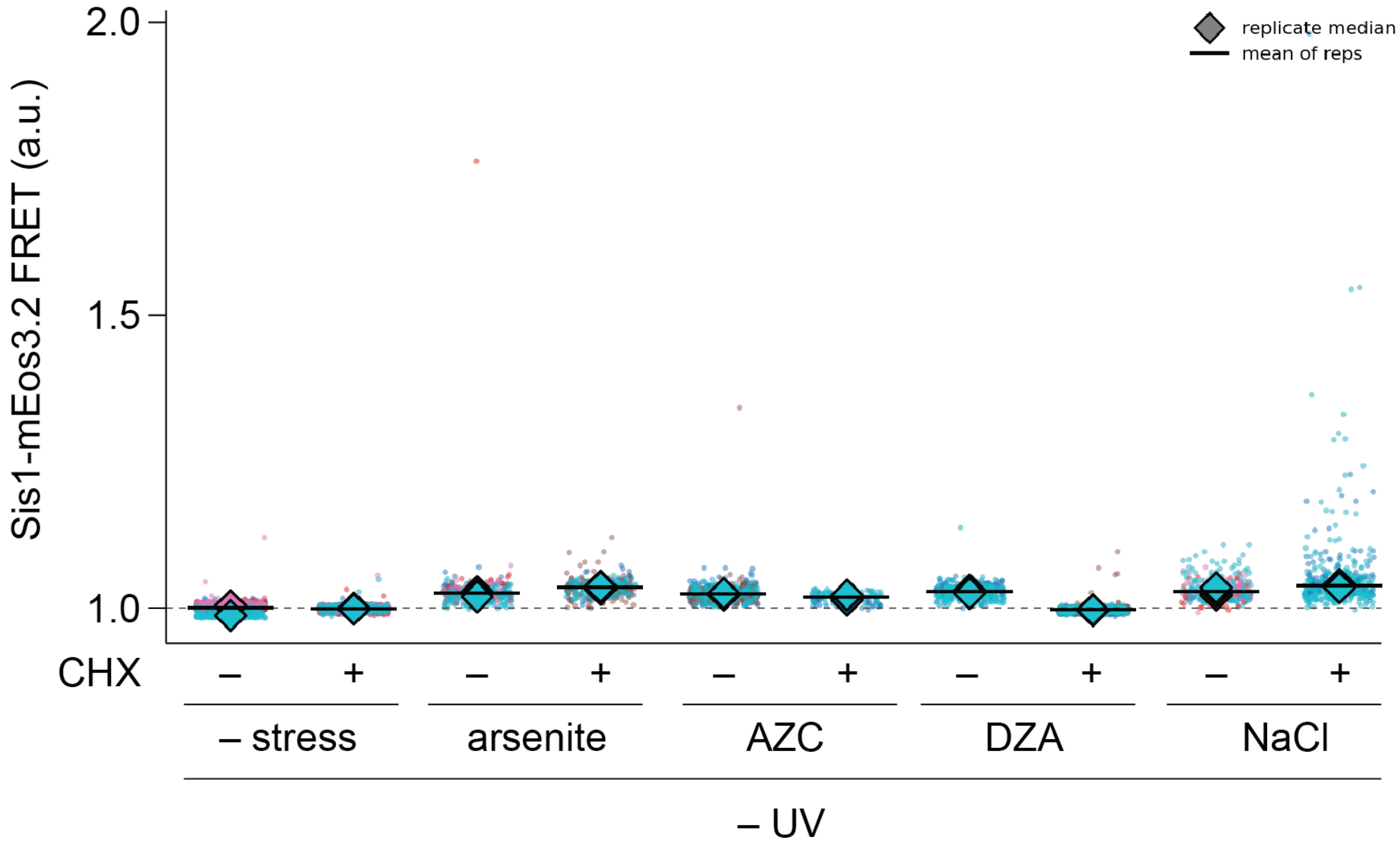


***Figure S4. Non-photoconverted (−UV) controls for the Sis1-mEos3.2 FRET measurements.*** *FRET signal in Sis1-mEos3.2 cells that were not UV-irradiated (−UV), acquired in parallel with the +UV data in Fig. 4 across the stress panel: unstressed (−stress), arsenite, AZC, DZA, and NaCl, each without and with 50 µg/ml cycloheximide (CHX). Without the 405 nm photoconversion pulse no acceptor pool is generated, so these samples report only background bleed-through and thus set the floor of the assay. FRET remains flat near this baseline in every condition, confirming that the stress-dependent signal in Fig. 4E reflects photoconversion-dependent Sis1 self-association. Superplot: small points, individual cells; diamonds, per-replicate medians; black bars, mean of replicate medians.*

bioRxiv preprint doi: https://doi.org/10.64898/2026.07.30.740154; this version posted July 30, 2026. The copyright holder for this preprint (which was not certified by peer review) is the author/funder, who has granted bioRxiv a license to display the preprint in perpetuity. It is made available under a CC-BY 4.0 International license.

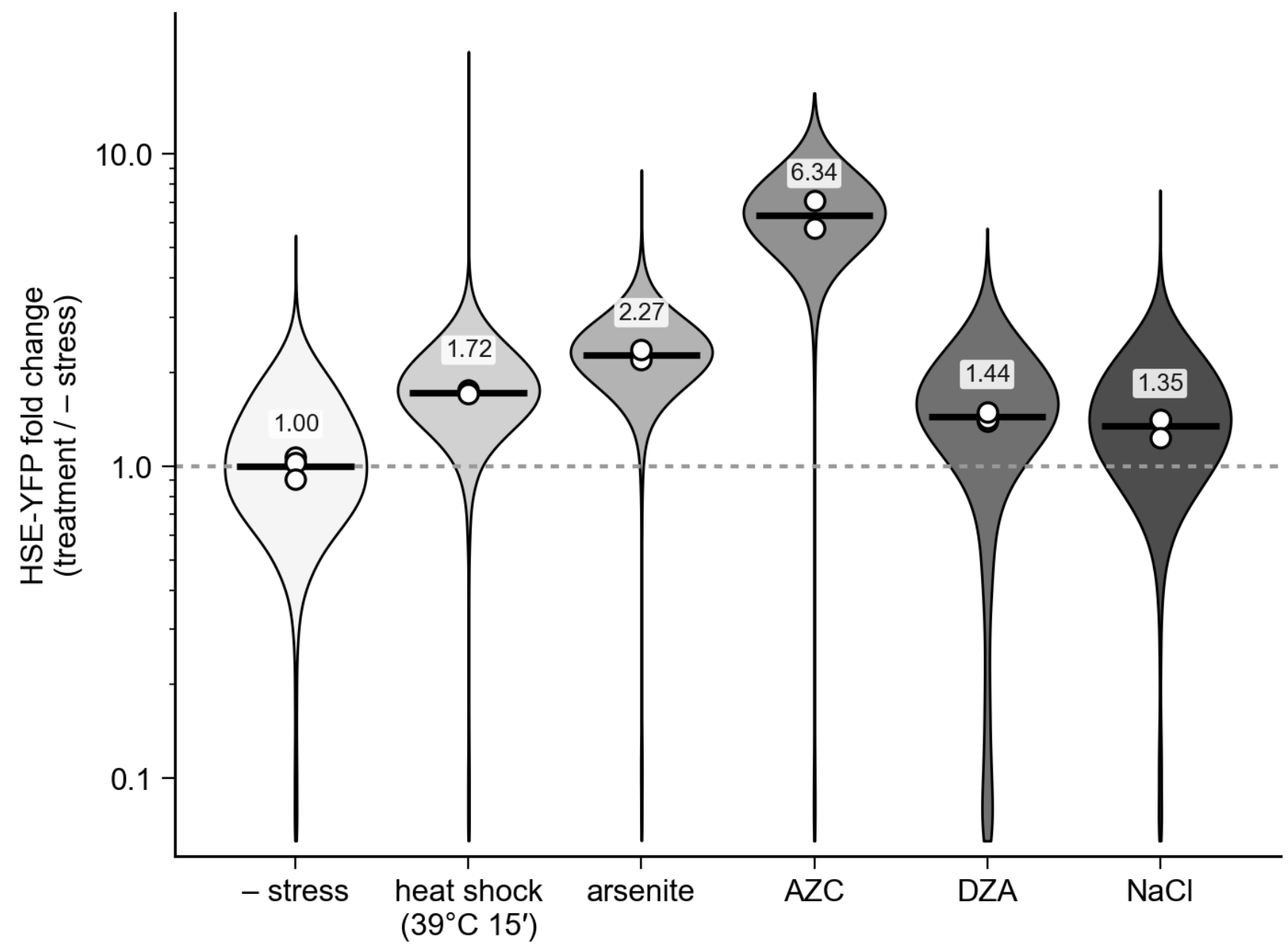


***Figure S5. HSE-YFP reporter registers AZC as a strong HSR inducer.*** *Flow cytometry of cells carrying an HSE-YFP transcriptional reporter (10,000 cells per condition). Per-cell reporter fluorescence (FITC), normalized to side scatter, is expressed as fold change relative to the median of unstressed (— stress) cells and plotted on a log scale. Violins, single-cell distributions pooled from three biological replicates; black bars, population medians (values above each violin); open circles, per-replicate medians; dashed line, no change. Stress conditions: heat shock, 39 °C, 15 min; arsenite, 1 mM, 1 h; AZC, 5 mM, 3 hrs; DZA, 25 µg/mL, 1 h; NaCl, 1 M, 30 min.*

bioRxiv preprint doi: https://doi.org/10.64898/2026.07.30.740154; this version posted July 30, 2026. The copyright holder for this preprint (which was not certified by peer review) is the author/funder, who has granted bioRxiv a license to display the preprint in perpetuity. It is made available under a CC-BY 4.0 International license.

# Supplementary Table

***Table S1. Yeast strains and plasmids used in this study.***

| Strain | Description | Relevant genotype (modifications to W303 MATa) | Figure(s) |
|---|---|---|---|
| DPY2167 | Orphan-RP / nucleolar imaging / Sis1 | *SIS1-mVenus::HIS, Rpl26a-Halo (CRISPR), mScarlet-Nsr1 (CRISPR)* | Fig. 1, 2 |
| DPY1846 | Halo-Ssa1 (Hsp70) | *SIS1-mVenus:HIS3, NSR1-mScarl:kanMX, HaloTag-SSA1::LEU* | Fig. 1, 2; S2 |
| DPY2266 | Hsf1 / Med15 / Rpb3 imaging | *HSF1-mVenus::HIS3, MED15-mCherry::hphMX, RPB3-mTFP1::kanMX* | Fig. 1, 2; S2 |
| DPY2122 | Sis1-mEos3.2 FRET biosensor | *SIS1-mEos3.2::kanMX* | Fig. 4; S4 |
| DPY2162 | Sis1 anchor-away + Hsf1 | *tor1-1, fpr1Δ::NAT, RPL13A-2×FKBP12::TRP1, SIS1-FRB-GFP::HIS3, HSF1-mKate::URA3, HaloTag-SSA1::LEU2* | Fig. 5 |
| DPY1298 | Sis1 anchor-away (RNA-seq) | *tor1-1, fpr1Δ::NAT, RPL13A-2×FKBP12::TRP1, SIS1-FRB-GFP::HIS3* | Fig. 3, 5 |
| DPY144 | HSE-YFP reporter | *HSE-mVenus::LEU2* | Fig. S5 |
